# Comparison of methods for cancer stem cell detection in prognosis of early stages NSCLC

**DOI:** 10.1038/s41416-024-02839-9

**Published:** 2024-09-20

**Authors:** Boutaîna Chandouri, Thomas Naves, May Yassine, Léa Ikhlef, Jérémy Tricard, Alain Chaunavel, Zeinab Homayed, Julie Pannequin, Nicolas Girard, Stéphanie Durand, Vincent Carré, Fabrice Lalloué

**Affiliations:** 1 UMR INSERM 1308 CAPTuR, Faculty of Medicine, University of Limoges, Limoges, France; 2 Carcidiag Biotechnologies company, Guéret, France; 3 Thoracic and Cardiovascular Surgery Department, Limoges University Hospital Center, Limoges, France; 4 Department of Pathology, Dupuytren University Hospital, Limoges, France; 5 IGF, Univ. Montpellier, CNRS, INSERM, Montpellier, France; 6 Thorax Institute Curie Montsouris, Institut Curie, Paris, France; 7 UVSQ, Paris Saclay University, Versailles, France

**Keywords:** Tumour biomarkers, Non-small-cell lung cancer

## Abstract

**Background:**

Despite advances in diagnosis and treatment in lung cancer, therapies still fail to improve patient management due to resistance mechanisms and relapses. As Cancer stem cells (CSCs) directly contribute to tumor growth and therapeutic resistance, their clinical detection represents a major challenge. However specific and additional CSC markers lack. Thus, our aim was to achieve selective detection of CSCs with specific glycan patterns and assess the CSCs burden to predict the risk of relapse in NSCLC tumors.

**Methods:**

The lung CSCs detection and sorting with a lectin MIX were assessed and compared to CD133 in vitro. Then, its putative role as CSC biomarker was evaluated in vivo and its clinical significance on 221 NSCLC patients.

**Results:**

We showed a significant CSCs enrichment in the MIX+ sorted fraction compared to CD133+ cells and confirmed its high tumorigenic capacity. The MIX prognostic value on the overall survival from early stages patients was validated suggesting its potential for detecting CSCs directly linked to tumor aggressiveness.

**Conclusion:**

The MIX could be more relevant for detecting and sorting CSCs than CD133. Moreover, its prognosis value could enable clinicians to better classify early-stage patients at high risk of relapse in order to tailor therapeutic decisions.

## Introduction

Lung cancer is the leading cause of cancer-related deaths worldwide, where non-small cell lung cancer (NSCLC) remains the most common subtype [1]. Despite advances in diagnosis and treatment, chemotherapy and targeted therapies have reduced efficacy due to heterogeneous resistance mechanisms. Lung cancer stem cells (CSCs) and significant heterogeneity are observed even in early-stage NSCLC [2, 3]. Following lung surgery, the absence of targeted treatments requires long-term follow-up to prevent relapse [4]. Therapeutic resistance remains a major concern, leading to high recurrence rates and poor survival outcomes rate within 2 years and poor 5-year survival outcomes (15% and 20% respectively) [5].

Growing evidence suggests the presence of CSCs in various tumor types even after treatment, and contribute to therapy resistance and disease relapse [6]. These CSCs share characteristics with normal stem cells including self-renewal but also play a distinct roles in tumor initiation, progression, aggressiveness and relapse [7]. Potential lung CSCs markers include surface biomarkers (CD133, EpCAM), nuclear transcription factors (Nanog, Oct4, Sox2), and the enzyme ALDH1A1 [8]. Identifying and quantifying CSCs in tumors holds promise for advancing therapy and improving prognostic assessment in lung cancer patients.

Existing CSCs markers lack specificity and reliability, also current detection and isolation techniques have limitations [9, 10]. There is a demand for CSCs-specific markers compatible with clinical techniques such as immunohistochemistry, to improve CSCs-based diagnostics and treatment strategies.

Furthermore, it has been recognized for many years that post-translational changes could be relevant markers of stem cells that disappear during the differentiation process [11, 12]. Thus, the importance of glycosylation changes in the stem cell marker CD133 during CSCs differentiation has been already underlined. While CD133 protein is present in both CSCs and differentiated tumor cells, the AC133 epitope is selectively masked and expressed only in CSCs [12]. However, the implementation of conventional methods to characterize CSCs glycosylation biomarkers in clinical practice remains challenging.

We previously developed a new approach to detect CSCs from a heterogeneous tumor cell population. This original methodology employs a combination of biotinylated plant lectins (UEA-1 and GSL-I) that selectively recognize glycan patterns expressed exclusively by CSCs. The significance of this new method for detecting CSCs has been demonstrated for the first time in colon cancer [13]. In this latter, patients with high ColoSTEM staining have a poor prognosis, with a significant reduction in 5-year overall survival (OS) even during the early stages of the disease. ColoSTEM staining outperforms the standard Oct-4 marker in distinguishing CSCs from non-stem tumor cells and healthy cells, suggesting its effectiveness in predicting tumor aggressiveness and therapeutic response in colon cancer patients. In the light of these findings, the development of an approach able to detect CSCs based on their glycosylation status might afford a relevant prognostic tool in lung cancer.

Here, we showed the detection of CSCs in NSCLC using a similar approach with a specific combination of lectins (MIX) recognizing glycosylated pattern exposed on cell surface. First, we compared the properties of Mix+ cells and CD133+ cells to demonstrate that combination of lectins enables isolating different cells from the CD133+ fraction related to CSCs. Subsequently, we analyzed the properties of Mix+ cells in different lung adenocarcinoma cell lines and compared their properties to cell subpopulations sorted by CD133. Our aim was to determine if cell sorting based on MIX was more efficient in isolating CSCs compared to CD133. In parallel, we reported the in vitro and in vivo validation results and analyzed the clinical relevance of CSCs detection at different stages by establishing the correlation between MIX staining and both overall survival (OS) and relapse free survival (RFS). Thus, we have validated CSCs-specific glycosylated motifs as biomarkers for predicting the aggressiveness and prognosis of lung cancer in early stages.

Unlike CD133+ cells, this new approach which enables of accurately detecting CSCs might help clinicians to better stratify early-stage patients with a high risk of relapse and to improve personalized patient management.

## Materials and methods

### Cell culture

The human NSCLC cell lines A549, H1975 and PC9 were obtained from the American Type Culture Collection (ATCC®, France) and were tested mycoplasma off. Cells were maintained in DMEM GlutaMAX^TM^ medium (Thermo Fisher, France) supplemented with 10% FBS (Thermo Fisher) and 1% penicillin/streptomycin (Thermo Fisher, France).

For 3D culture, a defined medium was used, composed of 1X DMEM/F12 medium (Thermo Fisher, France) supplemented with 1X B27 (Thermo Fisher, France), 10 ng/mL FGF (Peprotech, France), and 20 ng/mL EGF (Peprotech, France). Cells were cultured at 37 °C with 5% CO_2_ and 95% humidity.

### Patient tumor lung tissues

The study involved two patient cohorts. The first cohort, from Lyon University Hospital (Hospices Civils of Lyon, HCL), comprising 151 patients (100 males, 51 females) with an average of 63.15 years (+/−0.8), including 101 early-stage (stages I and II) and 50 late-stage (stages III and IV) patients. The patients of Lyon University Hospital were collected under broad consent and provided by the Lung Biobank from HCL (agreement Number: R4-P1-ST1.1.CRB-HCL). The second, from AMSBIO (AMS Biotechnology, Abingdon, UK), included 70 patients with an equal gender distribution and an average age of 62 years (+/−1.2), with 36 early-stage and 24 late-stage patients (Table 1).

**Table 1:** Clinicopathological characteristics of 221 patients with NSCLC.

Lung adenocarcinoma		Cohort 1 (Curie Institute)	Cohort 2 (AMSBIO)	Whole patients
Cohort size	*n*	151	70	221
Sex	Male	100	35	135
	Female	51	35	86
Age	Mean (+/−SEM) (yrs)	63.15 (+/−0.8)	62 (+/−1.2)	62.8 (+/−0.7)
	Median (min–max) (yrs)	63.3 (39.7–87.6)	61.5 (30–84)	63.1 (30–87.6)
Age class	≤60 yrs	54	34	88
	>60 yrs	97	36	133
Stage	I	69	26	95
	II	32	10	42
	III	44	23	67
	IV	6	1	7
	n.a	/	10	10
Stage	Early (I + II)	101	36	137
	Late (III + IV)	50	24	74
Vital status	Alive	78	20	98
	Dead	73	50	123
*LungSTEM* MIX	Low staining	61	25	86
	High staining	85	43	128
	n.a	5	2	7
Overall survival (OS)	Median OS (months)	71.2	39	57
	(95% confidence limit)	(57.9–n.a)	(29–55)	(49.3–77.8)

*n.a* not available.

### Application of a method based on lectin combination for detecting CSCs in NSCLC

The detection and sorting of CSCs were performed with a MIX of lectins enable to recognize specific glycosylation profile. This MIX and method were firstly described in two patents (FR20150061763 - WO2016FR53196; WO2016FR53197- FR20150061764) regarding colon cancer. These previous results and patents allowed to detect colon CSCs with a specific lectin Mix enabled to identify CSCs glycosylation pattern. Preliminary experiments have permitted to demonstrate the clinical relevance of this method in CSC detection from colon cancer patients. Its ability to predict tumor aggressiveness was determined as well as its prognosis value regarding therapeutic response [13]. This method was adapted to characterize lung CSCs and resulted in the addition of two new patents (FR20170055137; FR20170055139 - WO2018FR51280) based on a specific ratio of lectin mix (UEA-1 and GSL-I). In this study, this last method was applied for detecting the CSCs in NSCLC in vitro and in vivo and for validating the clinical potential on a large lung cancer patient cohort.

### Cell sorting of LungSTEM fluorescent cells

A suspension of 2.10^7^ cells/mL were sorted by FACS (BD FACSAria™ III sorters for Single-cells sorting) based on LungSTEM MIX. For clonogenic assay, a single cell was placed per well in ultra-low attachment 96-well plates (Falcon Corning brand, France). Dying cells were excluded from sorting by adding Propidium iodide (PI).

For flow cytometer analysis, sorted cells were labeled either extracellular anti-CD133-APC (C15190, Beckman Coulter) or anti-EpCAM-APC (130-109-764, Miltenyi, France) antibodies. Then, cells were incubated 30 min, washed in PBS, fixed 10 min in 4% PFA and analyzed with a CytoFLEX (Beckman Coulter, USA). Data analysis were performed using Kaluza software (v2.1, Beckman Coulter, USA).

### Indirect magnetic cell sorting

MACS sorting was performed from 2.10^7^ cells/mL using the CELLection™ Biotin Binder kit (Invitrogen-ThermoFisher Scientific, France) according to the manufacturer’s instructions, by using 10 µg of either the MIX or the AC133 biotinylated antibody (Miltenyi Biotech, France). For the clonogenic assay, sorted cells were seeded in ultra-low attachment 96-well plates (Falcon Corning brand, France) at decreasing cell densities (1000, 100, 10, and 1 cell) to evaluate their clonogenic potential.

### Clonogenicity assay

In each experimental condition sorted cells were seeded in triplicate in a defined medium. Weekly, 50 microliters of the medium were added to each well, and this process continued for a period of 4–8 weeks. The quantification of sphere formation was performed per well and per condition, employing an optical microscope (Olympus CKX53, Life science, Waltham, Massachusetts) at a magnification of ×100. The spheroids were consistently imaged at regular intervals, precisely every 7^th^ day (Day+7). The measurement of spheroid size was accomplished using ImageJ software.

### Cell viability assay

For each condition, 1500 cells were sorted and seeded in a 96-well plate. After 24 h of cell adhesion, cells were treated or not, with increasing doses of Cisplatin (0–200 µM). Following 72 h of incubation, the Cell Viability Kit reagent (Promega, WI, UA) was added according to the manufacturer’s instructions and luminescence was measured with EnSpire® Multimode Plate Reader (PerkinElmer, USA). The IC_50_ was determined graphically with GraphPad Prism 7.04 software.

### RNA extraction and RT-qPCR

Total RNAs were extracted with Quick-RNA Microprep Kit (Zymo R1051, USA). cDNAs synthesis were performed with the cDNA Archive kit (Applied Biosystems, MA, US) and qPCR were performed with SensiFAST Probe Hi-ROX kit (Bioline, London, UK) on a QuantStudio 5 system (Thermo Fisher, France). The expression level of stemness-related genes, *NANOG* (Hs02387400_g1; Thermo Fisher, France), *POUF5* (OCT4) *(*Hs00999634_gH; Thermo Fisher, France), *PROM1* (CD133) (Hs01009259_m1; Thermo Fisher, France), and *SOX2* (Hs01053049_s1; Thermo Fisher, France) were normalized to housekeeping genes, *ActB* (Hs01064291, Thermo Fisher, France) and *GAPDH* (Hs02786624, Thermo Fisher, France) expression and quantified by the ΔΔCt method.

### Western-blot

Cells were lysed using RIPA buffer (Thermo Scientific™, France), centrifuged and protein concentration were determined using a Bradford protein assay (BioRad). Samples were separated on SDS-PAGE, transferred to a PVDF membrane (GE Healthcare), and blocked for 1 h in 5% BSA-PBS before being incubated with primary antibodies, including anti-CD133 (130-113-107; Miltenyi Biotec, France), anti-EpCAM; (130-110-997; Miltenyi Biotec, France), anti-Oct4 (130-109-764; Miltenyi Biotec, France), anti-SLUG (C19G7; Ozyme, France), anti-Snail (C15D3; Ozyme, France) and anti-ZEB1 (3396; Ozyme, France), overnight at 4 °C. Then, membranes were incubated with secondary antibodies (Dako Cytomation) for 1 h and were revealed using a G-Box (Syngene, Fisher Scientific, France).

### Migration assay

The sorted cells were seeded to reach full confluence within 24 h. The following day, wounds were created with a WoundMaker^TM^ (Sartorius, Goettingen, Germany) according to manufacturer’s instructions. Then, 100 µl of culture medium was added to the wells. The cell migration was monitored with Incucyte (Sartorius, Goettingen, Germany) every 2 h for 48 h with Rapid Red Nuclight (NC1404054, Sartorius, Goettingen, Germany). Wound healings were measured with IncuCyte software (Incucyte 2022 Rev1 software, Sartorius).

### Invasion assay

Spheroids were embedded in 100 µL of Matrigel (Corning, 356255, USA) at 100 µg/mL in a 96-well plate and 100 µL of culture medium was added. Spheroids were imaged daily with optical microscope (Olympus CKX53) at a magnification of ×100. Matrigel invasion were measured with the Fiji Macro analysis program with ImageJ software [14].

### In vivo tumorigenic assay

After cell sorting based on MIX, MIX+ and MIX− cells were injected in decreasing numbers of 5000, 500, and 50 cells into 5 different mice per condition. Tumor growth and size were monitored over time. All animal experiments were approved by the French Agriculture and Forestry Ministry (APAFIS number: 2019071709325396 #21596).

### Immunohistochemistry

Immunohistochemistry staining was performed on 4 µm paraffin-embedded histological sections using the Leica Bond Max automatic staining platform (Leica, France). The sections were stained with a pre-diluted MIX in diluent and detected with the Bond Intense R Detection kit and Bond Polymer Refine Red Detection kit (Leica, France). Nuclei were counter-stained with hematoxylin, and the slides were examined with the NanoZoomer RS 2.0 Hamamatsu (Hamamatsu Photonics, Massy, France) [13]. MIX staining resulted in brown staining of membranes and/or cytoplasm. A scoring method based on a staining threshold of 30% categorized patients as “Low staining” or “High staining”.

The MIX staining and its correlation with different clinical parameters were achieved on lung tumor obtained from HCL and AMSBIO (Table 1). Additionally, the MIX effectiveness of the MIX in recognizing only CSCs, we used 62 non-tumor lung tissues from Tissue Microarray (TMA) as the MIX-negative controls, which were provided by AMSBIO (AMS Biotechnology Europe, UK). In this way, the sensitivity and specificity were evaluated by comparing the MIX staining on 62 non-tumor tissue samples with NSCLC samples using the ROC (Receiver Operating Characteristic) curve.

The proliferation rate was assessed with Ki67 antibody (clone 30-9, Ventana-Roche Medical Systems) following protocols provided by antibody manufacturers in the pathology departments of Limoges University Hospital. A ratio of Ki67-positive and negative cells to the total cell count was calculated.

### Statistical analysis

Statistical software used in this study were Prism 7 (GraphPad, USA) or R environment (version 4.0.3). Statistical analysis of in vitro and in vivo experiments was made using t-test for two groups comparison and one-way ANOVA test, for three groups comparison variables. The normal distribution was checked using Shapiro–Wilk test. The prognostic value of each parameter for the outcome (overall survival and relapse-free survival) was assessed using Kaplan–Meier method and log-rank test (Mantel–Cox). For each variable, hazard ratio (HR) was estimated using a univariate Cox model and expressed with their 95% confidence interval (95% CI). Multivariate analysis was carried out using a Cox regression model on single features identified by the univariate Cox modeling. Survival analysis were performed in R using survival and *survminer* packages. The proportional hazards assumption for Cox regression model fit was verified using *cox.zph* function of survival package. A *p*-value below 0.05 was considered as significant.

## Results

### Discrimination of cells with stemness properties from a heterogeneous tumor cell subpopulation based on specific glycosylation pattern

**Fig. 1:**
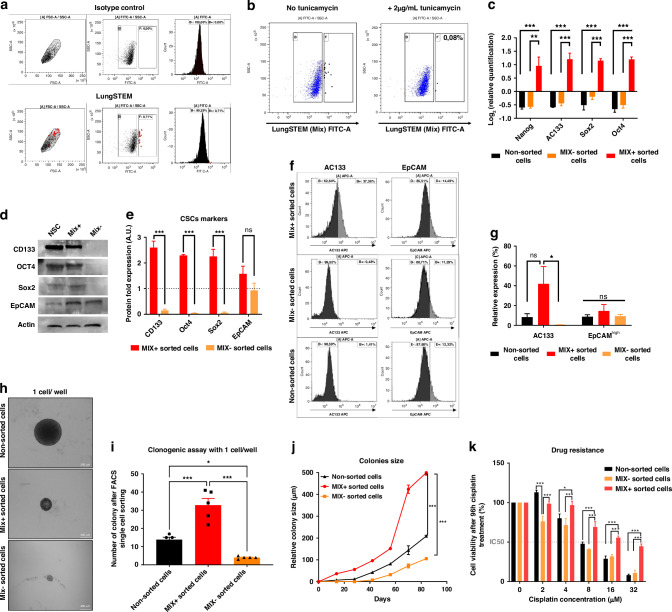
In Vitro characterization of LungStem kit (MIX of lectins) efficiency in a NSCLC cell line (A549). **a** Representative FACS dot plots (top) showing the recognition of glycosylated patterns by the isotypic (diluent) control condition. Bottom, expression of glycosylated patterns detected by the LungSTEM kit (MIX). **b** Representative FACS dot plots showing the recognition of glycosylated patterns by the LungSTEM without (at left panel) and with addition of 2 µg/mL of Tunicamycin. **c** Representing mRNA expression levels of cancer stem cell genes (AC133, Nanog, Oct4 and Sox2) in different sorted sub-population (MIX+, MIX− and Non-sorted cells). **d** Western Blot shows the cancer stem cell related proteins expression in each sorted sub-population (MIX+, MIX− and non-sorted cells). **e** Western Blot quantifications normalized to non-sorted cells out of three replicates. **f** Representative flow cytometry dot plots of relative expression of AC133 and EpCAMhigh after FACS single cell sorting on LungSTEM compared with non-sorted cells. **g** Histogram representing EpCAMhigh and AC133+ percentages analyzed by Flow cytometry within MIX+, MIX− and Non-sorted cells. **h** Clonogenic capacity after FACS single cell sorting. Representative illustrations are depicted (magnification ×100). **i** The mean of number of colonies formed from MIX+, MIX− and non-sorted cells after FACS single sorting cells (After 60 days of incubation). **j** Relative colonies sizes of MIX+ and MIX− sorted cells compared with control non-sorted cells after FACS single cell sorting. The spheres’ size was monitored by taking pictures every D+7 for 80 days. **k** Histogram representing drug resistance to Cisplatin of MIX+, MIX− and non-sorted cells after FACS cell sorting with 1500 cells seeded per well, after 5 replicates. Results are represented as mean ± SEM, ns for not significant result, *p-value < 0.05, **p-value < 0.01, ***p-value < 0.001 using one-way ANOVA test and Student’s *t* test (*n* = 3 to *n* = 5 experiments).

The effectiveness and reliability of this new lectin combination to discriminate CSCs based on their glycan signature from whole cancer cell population were tested. For demonstrating that CSCs could be sorted with a specific MIX from A549 tumor cell line, we performed FACS analyses. Our results have shown that lectin mix conjugated to fluorescent marker only recognized around 1% of A549 cells (Fig. 1a). To confirm the specificity of lectin binding to glycan cell surfaces and eliminate false positives, we used the glycosylation inhibitor Tunicamycin, which impaired lectin labeling (Fig. 1b). Through single-cell sorting by FACS using MIX positivity, we analyzed CSCs-related transcripts, proteins and functional properties from A549 NSCLC cells. Transcriptomic analysis carried out by RT-qPCR confirmed that CSCs-related genes (Nanog, AC133, Oct4, EpCAM, Sox2) are significantly increased in MIX-positive sorted cells compared to MIX-negative cells or non-sorted cells (NSC) (Fig. 1c). Interestingly enough, compared to MIX-negative and NSC cells, CSC-related protein markers (Nanog, AC133, Oct4, EpCAM, Sox2) are significantly overexpressed in MIX-positive cells as showed in both western blotting (Fig. 1d, e) and flow cytometry (Fig. 1f, g). To determine if the proportion of MIX+ cells could change in EGFR mutated cells, we analyzed the percentage of MIX positive cells in both mutated cells lines, PC9 and H1975. Although the percentage of MIX positive cells were significantly increase with approximately 43% in H1975 expressing EGFR with activating mutations, L858R and T790M, this percentage remains close to A549 with 1% for PC9 which harbors an EGFR exon 19 deletion (Supplementary Fig. S1A). We confirmed that CSC markers, CD133, EpCAM and Sox2 are markedly elevated in MIX+ sorted cells from H1975 and PC9 compared to A549 suggesting that CSC markers are increased in EGFR-mutated cell line (Supplementary Fig. S1B, C). Previous results support this hypothesis demonstrating EGFR pathway deregulation promotes the emergence of stem like properties in non-small-cell lung cancer and is predictive of worse outcome to EGFR inhibition [15]. Since MIX+ cells expressed various CSC-related markers, we chose this one to better analyze hallmarks of CSCs. Then, we conducted clonogenicity and drug resistance tests from FACS single cell sorting in 96 wells plate. As attempted, MIX-positive A549 cells, characterized by their unique glycan signature, exhibited a significantly higher self-renewal capacity (*p* = 0.002) compared to negative and unsorted cells. This was significantly evident through the enhanced number and size of spheroids (*p* < 0.01) derived from MIX-positive-sorted cells (Fig. 1h–j). Likewise, treatment with increasing doses of Cisplatin showed significantly higher IC50 (*p* < 0.001) in the MIX-positive-sorted cells compared to both non-sorted and negative cells suggesting that MIX-positive cells present these resistance abilities own to CSC (Fig. 1k). Similar observations have been done in both other cancer cell lines in which the size of tumorospheres derived from MIX+ cells was significantly increased (*p* = 0.003) compared to those formed by MIX negative or non-sorted cells (Supplementary Fig. S1D–G*)*. Similarly, we confirmed that cisplatin treatment induces similar effect to those observed with A549 on PC9 and H1975 cells. In the latter, cell viability measured by IC50 were significantly higher in MIX+ cells (*p* = 0.0016) compared to the other subpopulations, suggesting that MIX+ cells are less sensitive to Cisplatin treatment than non-sorted and MIX negative cells (Supplementary Fig. S1N, O).

**Fig. 2:**
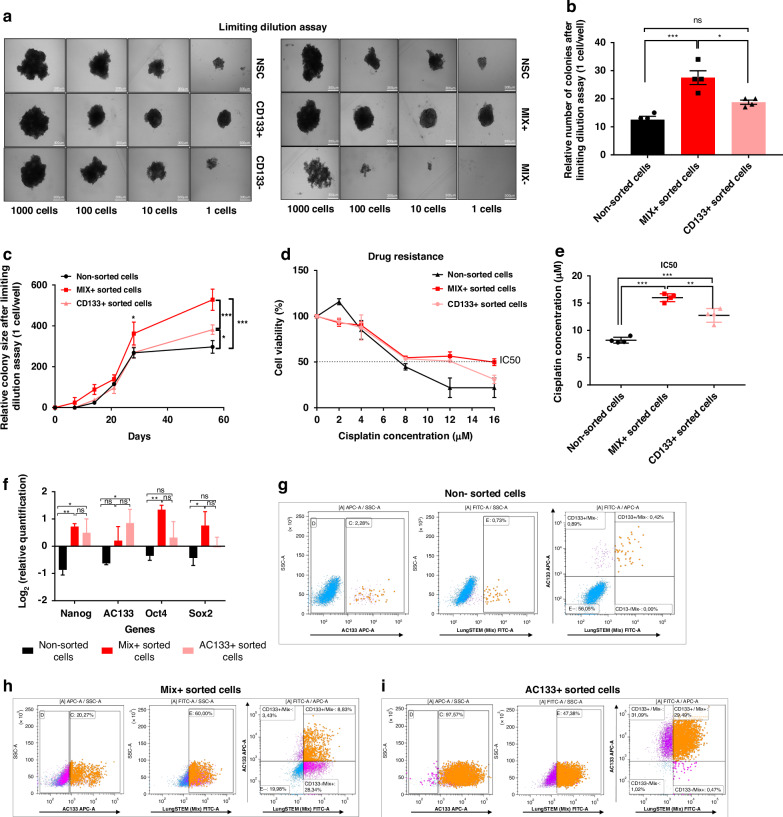
In Vitro characterization of MIX efficiency in lung CSCs detection compared to CD133. **a** Clonogenic capacity after Limiting Dilution Assay. Representative sphere forming ability in different sorted sub-population are depicted, from AC133 (AC133+ and AC133− sorted-cells compared with Non-sorted cell (NSC) or lungSTEM MIX (MIX+ and MIX− sorted cells compared with Non-sorted cell (NSC), according to seeded cell densities (1, 10, 100, 1000 cells/well) (magnification, ×100). **b** Histograms represent the mean of sphere number formed after Limiting Dilution Assay with ie. NSC or MIX+ sorted cells or CD133 positive sorted cells in the condition with 1 cell/well. **c** Relative colony size in condition of 1 cell/well following Limiting Dilution Assay in different sorted sub-population (AC133+, MIX+ and NSC). **d** Response to increasing concentrations of cisplatin treatment after MACS cell sorting with AC133 or with LungSTEM MIX to assess drug resistance in MIX+, AC133+ and Non-sorted cells. **e** Representation of the average IC50 of Cisplatin (µM) in the different sorted subpopulations: MIX+, AC133+ and Non-sorted cells. **f** Analysis of CSCs genes levels (Nanog, AC133, Oct4, Sox2) in different sorted sub-population (MIX+, AC133+ and Non-sorted cells). **g** Representative FACS dot plots showing the basal expression of AC133 (at left), LungSTEM MIX (at middle) and coexpression (at right) in Non-sorted cells. **h** Representative FACS dot plots showing AC133 expression (at left), LungSTEM MIX expression (at middle) and coexpression of AC133 and MIX (at right) in Mix+ sorted cells. **i** Representative FACS dot plots showing AC133 expression (at left), LungSTEM expression (at middle) and coexpression of AC133 and MIX (at right) in AC133+ sorted cells. All results are represented as mean ± SEM, ns *p*-value indicate not significant result, *p-value < 0.05, **p-value < 0.01, ***p-value < 0.001 using one-way ANOVA test (*n* = 4 experiments).

In parallel, these findings suggest that MIX-positive cells share similar properties than CSCs sorted by CD133 (AC133). Given that a reduced percentage of MIX+ cells expressed AC133 (8.83%), we compared cancer stem cell-like properties between MIX-sorted and AC133-sorted cells in A549, H1975 and PC9 cells (Fig. 2g, h, i and Supplementary Fig. S2F, G). We observed that CD133−/MIX+ cells display respectively 40% (PC9) and 10% (H1975) of whole cell population whereas the percentage of CD133+/MIX+ cells is comparatively reduced to 20% and 1% in each cell line (Supplementary Fig S2F, G). Thus, we concluded that CD133 expression was reduced in MIX positive cells subpopulation whatever the adenocarcinoma cell lines. Therefore, we performed a Limiting Dilution Assay to challenge the self-renewal capacities between AC133++ and Mix sorted cells as this latter are composed of less AC133+ cells. Strikingly, we found that the Mix+ fraction had a significantly higher capacity to form spheroids (*p* = 0.0117) compared to the other fractions (Fig. 2a, b). Interestingly enough, spheroids formed by MIX+ cells exhibited a significant increase in size (*p* = 0.003) compared to those formed by AC133+ cells (Fig. 2c). We confirmed in both EGFR mutated cell lines, H1975 and PC9, that MIX-positive cells formed more and larger colonies compared to the CD133− fraction after analyzing AC133/MIX sorted cells, respectively *p* = 0.0044 and *p* = 0.0463 (Supplementary Fig. S2A–C). Resistance to cisplatin treatment has also been verified at various concentrations on both cell subpopulations AC133 or MIX+ sorted cells. The MIX+ A549 subpopulation cells exhibited a significantly higher IC50 (*p* = 0.0016) compared to the AC133+ subpopulation, indicating that MIX+ cells have greater resistance to Cisplatin treatment than AC133+ cells (Fig. 2d, e). Likewise, MIX+ cells demonstrated significantly greater resistance to treatment compared to AC133+ cells in the H1975 cell line (*p* = 0.0385), and a trend towards increased resistance in PC9 (*p* = 0.1075) (Supplementary Fig. S2D). Since stemness-related genes were found to be significantly overexpressed in both MIX+ and AC133+ sorted cells when compared to MIX−, AC133−, and unsorted cells, these results suggest that MIX+ cells present stemness properties as observed with AC133+ cells (Fig. 2f). The enrichment of CSC markers SOX2 and AC133 was notably similar in both EGFR mutated cells lines for AC133+ and MIX+ fractions contrarily to Nanog which is predominantly increased in MIX+ cells (Supplementary Fig. S2E).

Thus, our data revealed that the MIX is more selective for lung CSCs compared to the AC133 antibody-based sorting method. These results demonstrate that the MIX significantly highlight a fraction of cells with CSC hallmarks.

### MIX-sorted cells express markers and functional properties of epithelial to mesenchymal transition (EMT)

**Fig. 3:**
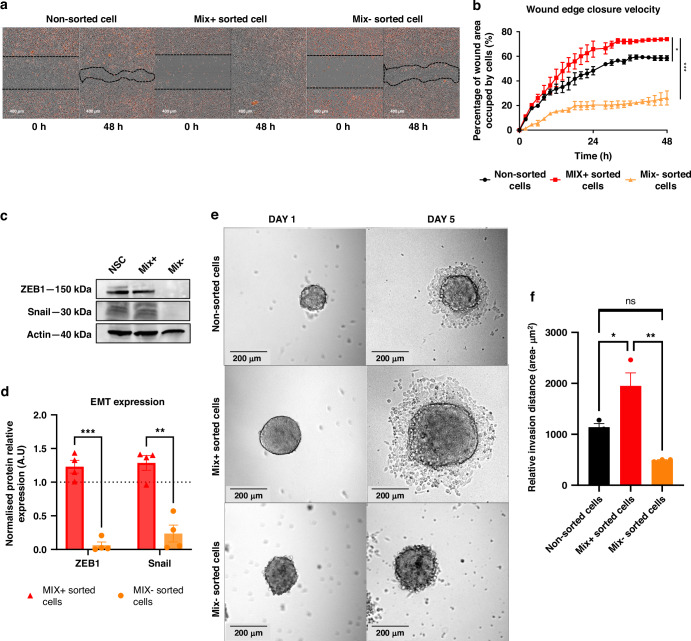
Implication of MIX+ stem like cells in the migration and invasion. **a** Representative illustrations of wound healing scratch assay are depicted using Incucyte 2022 Rev1 (magnification ×10). **b** Wound healing scratch assay for unsorted, MIX+ and MIX− sorted cells. Percentage of wound healing was measured using Incucyte 2022 Rev1 Software. **c** Western Blot shows Epithelial-mesenchymal transition (EMT) related proteins levels in each sorted sub-population (MIX+, MIX− and non-sorted cells). **d** Western Blot quantifications normalized to non-sorted cells out of three replicates. **e** Representative images of spheroids’ invasion capacities after 5 days of incubation in Matrigel coated inserts, in each sorted sub-population (MIX+, MIX− and non-sorted cells). **f** Histogram representing the relative invasion capacity of spheroids from each sorted subpopulation (MIX+, MIX− and unsorted cells). Spheroids from each subpopulation were embedded in Matrigel. Matrigel invasion was measured by deducting the total area from the central area, using the Fiji Macro analysis program. Some results are represented as mean ± SEM, ns not significant result, *p-value < 0.05, **p-value < 0.01, ***p-value < 0.001 using one-way ANOVA test (*n* = 3 experiments) and others Student’s *t* test (*n* = 4 experiments).

Since CSCs were also endowed with epithelial-to-mesenchymal transition, we analyzed the cells ability to migrate and invade. Hence, we performed wound healing scratch and invasion tests following FACS sorting cells based on MIX labeling. We found that, fraction expressing the glycan signature recognized by the MIX showed a significantly increased migration capacity compared with both MIX− (*p* < 0.001, orange curve) and unsorted fractions (*p* < 0.05, black curve) (Fig. 3a, b). These results were confirmed in the other two mutated cell lines, H1975 and PC9, where the MIX+ fraction also showed a significantly higher migration capacity compared to other fractions (*p* < 0.0001) (Supplementary Fig. S3A–D).

To strengthen these findings, we performed western blotting analysis to assess the expression of EMT-specific proteins such as Snail and ZEB1. Remarkably, we observed that MIX+ sorted A549 cells exhibited a significant upregulation of these markers (*p* < 0.001) (Fig. 3c, d). Similar results were observed in the PC9 (*p* = 0.003) and H1975 (*p* = 0.0009) lines (Supplementary Fig. S3E, F).

Additionally, spheroids previously obtained after FACS single cell sorting based on Mix staining were seeded on the surface of a Matrigel matrix to observe cell invasion. Notably, a distinct halo of invasive cells was observed surrounding the spheroids exclusively only in the MIX+ and non-sorted cells. As observed, the cell ability to invade and migrate was significantly concentrated in the MIX+ population (*p* = 0.0013) (Fig. 3e, f). These observations were also noted and quantified in the H1975 (*p* = 0.0009) and PC9 cells *p* = 0.0034) (Supplementary Fig. S3G, H, I, J).

Taken together, these functional characterization results highlighted that the MIX+ subpopulation exhibits CSCs hallmarks such as specific stemness markers, clonogenicity, drug resistance as well as invasion ability. These results would suggest that in vivo, MIX+ cells would present more aggressive behavior than another cell fraction.

### Tumorigenic potential of MIX+ sorted cells

**Fig. 4:**
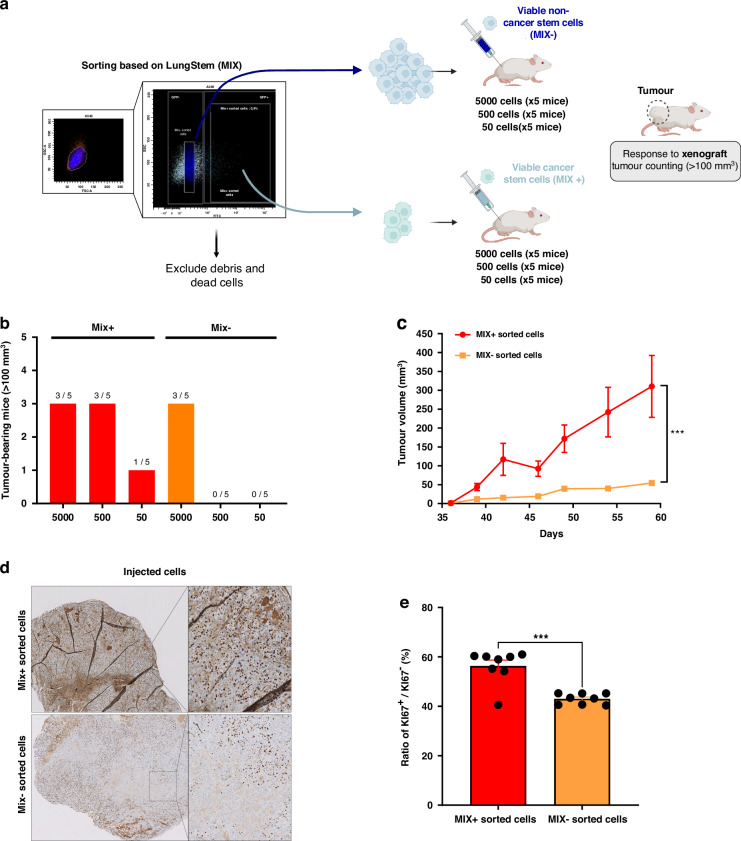
In Vivo tumorigenesis capacities of MIX positive cells efficiency in lung CSCs detection: tumorigenic potential of Mix+ and Mix− cells isolated from A549. **a** Illustration of the process followed after FACS-sorting on the LungSTEM (MIX) and injection to different mice. MIX+ sorted cells and MIX− sorted cells were subcutaneously transplanted into nude mice. **b** Illustration representing the number of tumors formed (tumor > 100 mm^3^) by mice according to the decreasing number of injected cells from each subpopulation (MIX+ and MIX−). **c** Tumors growth curve representing the evolution of tumor size after 500 cells injected in each mouse. It was monitored by the size from 1 to 60 days after the cells were injected into nude mice. The tumor size was the average of the vertical and horizontal diameters (*n* = 5 mice for each condition). **d** Representative illustrations of Ki-67 staining are depicted with higher magnification (×200). **e** Histogram representing Ki-67 staining measured in each subpopulation (MIX+ and MIX−) using the Fiji Macro analysis program on eight sections. All results are represented as mean ± SEM, ns not significant, *p-value < 0.05, **p-value < 0.01, ***p-value < 0.001 using Student’s *t* test.

To demonstrate that MIX+ cells could be related to CSCs, MIX+ tumorigenicity was analyzed in immunocompromised animal host. After FACS sorting, 45 nude mice were injected subcutaneously with different amounts of cells sorted either by MIX+, MIX− or unsorted dilutions, as shown in Fig. 4a.

The number of mice with tumors larger than 100 mm^3^ was counted sixty days after injection (Fig. 4a). Interestingly, among the 15 mice transplanted with the MIX− sorted cell subpopulation, only 3 mice developed tumors whereas 7 mice have tumors derived from MIX+ sorted cells (Fig. 4b). Strikingly, MIX+ cell subpopulation remains the sole condition to observe initiating tumor formation from the 500 and even 50 cells injected grafts (Fig. 4b). These results suggest that the tumorigenicity of MIX+ cells are higher than those of MIX−. Since a limited number of MIX positive cells (50 cells) are required to initiate tumors, we can conclude that MIX+ cells with their stem-like features would be more aggressive.

The analysis of the tumor volume from the different xenograft supports our initial findings. Indeed, MIX+ derived tumors had an average tumor volume 6 times greater than tumors derived from other cells (*p* = 0.0005). MIX+ sorted cells resulted in larger tumor volumes (300 mm^3^) compared to MIX− sorted cells (50 mm^3^). Importantly, MIX+ cells achieved a mean tumor volume exceeding 100 mm^3^ within 40–45 days after a 500-cells injection, whereas no tumors reached this size with MIX− (Fig. 4c). These results suggest that tumors obtained after injection of the MIX+ sorted subpopulation remains more aggressive than those obtained with the MIX− sorted subpopulation.

We assessed cell proliferation rates in the tumors using Ki67 staining. Consistent with expectations, the immunohistochemical analysis of Ki67 in tumor sections from both the MIX+ and MIX− subpopulations revealed a significant increase proliferation (*p* = 0.0013) in the MIX+ group compared to the MIX− group (Fig. 4d, e).

Altogether, these results would confirm in vitro findings and demonstrate that the sorted MIX+ subpopulation is enriched in CSCs due to their ability to initiate tumors even with a significantly reduced grafted cells number.

### Detection of NSCLC patients with MIX+ cells

**Fig. 5:**
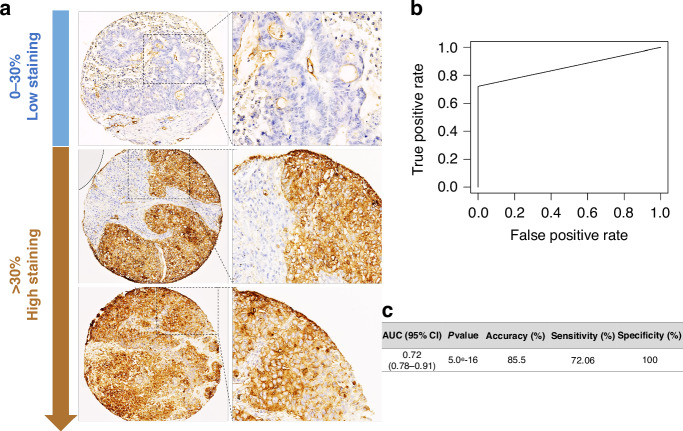
Validation of LungSTEM MIX staining efficiency in the detection of NSCLC patients. **a** Representative illustrations of LungSTEM (MIX) staining (in brown), as observed by IHC are depicted (magnification, 200×). **b** The Receiver Operating characteristic (ROC) curve of LungSTEM (MIX) and mainly performance parameters of the classification (Accurancy, sensitivity and specificity).

*The* in vitro and in vivo experiments revealed that MIX-positive subpopulation exhibits CSCs features and also could be related to Lung CSCs. In these conditions, we decided to study its clinical relevance in discriminating healthy tissue from lung tumor. We analyzed whether MIX-positive could accurately detect lung CSCs directly in tissue samples by IHC. The staining with MIX was performed on 221 tumor tissues from two different cohorts (from Lyon University Hospices and AMSBIO) (Table 1). Among the stained cell TMA, 86 were scored as MIX-Low (0–30% stained cell) and 128 as MIX-High (>30% stained cell) (Fig. 5a, Table 1). Independence between MIX staining and others clinical variables were confirmed by Chi-2 test (*vs* Sex: *p* = 0.17; *vs* Age class: *p* = 0.81; *vs* Stage, *p* = 0.33).

First, we evaluated the specificity and sensitivity of MIX staining using 70 tumoral lung tissues (36 early stage and 34 late stage) and 63 paired non-tumoral tissues. None of non-tumoral harboring positive-cells after MIX staining, indicating an excellent specificity (100%). After application of 30%-cutoff of MIX-staining, 19 tumoral tissues can be considered as false negative, reaching a sensitivity of 72.1% (Fig. 5b). ROC curve built from results of MIX staining on non-tumor and tumor lung tissue permit to establish that AUC is acceptable (AUC = 0.72; data not shown) and confirmed a good accuracy (86%, 95% CI: 0.78–0.91; *p* = 5 × 10^−16^; Fig. 5b). These results suggest that the MIX might be likely to discriminate CSCs from healthy stem cells. (Fig. 5b, c).

### NSCLC patients grading based on tumor aggressiveness and prognostic value of MIX+ staining

Secondly, we aim to assess whether the MIX-positive cells could predict patient relapse or survival prognosis. Overall survival (OS) analysis were evaluated by Kaplan–Meier curves and Cox regression models, according to MIX staining (MIX-Low and High) and others clinicopathological data, i.e. sex (men and women), age (< and ≥60 years old) and stage (early, I/II and late, III/IV). Noted that OS analysis has been performed on whole patients (*n* = 221) from HCL and AMSBIO cohorts (Table 1), while RFS has been conducted only on HCL cohort (*n* = 70), because AMSBIO failed onto available recurrence information.

**Fig. 6:**
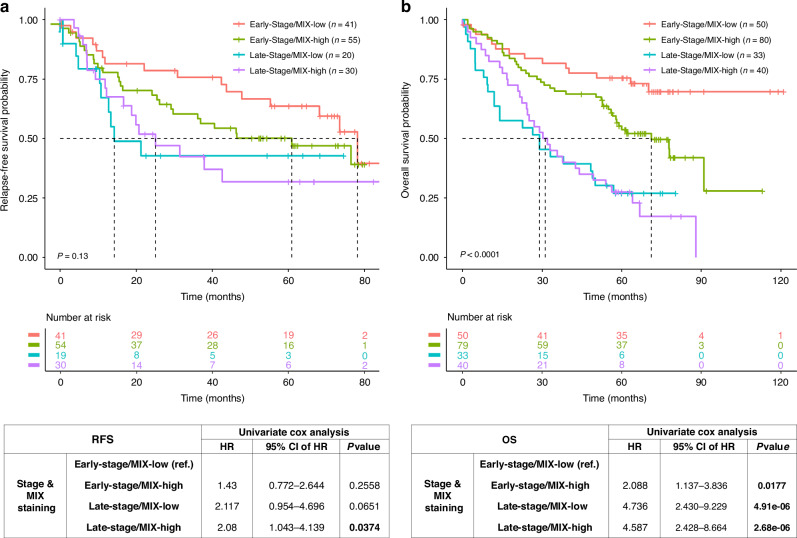
Prognosis value of MIX staining combined with stages on relapse-free survival and overall survival. Relapse-free survival (RFS) from Lyon University Hospital cohort (**a**) and overall survival (OS) from both cohorts (**b**) was analyzed according to pTNM stage (early stage and late stage) of NSCLC patients and LungSTEM (MIX) staining intensity (low/high). Kaplan–Meier curves are depicted according to MIX staining combined with lung adenocarcinoma stages. *P* value indicated in each panel correspond to log-rank test (Mantel–Cox) performed to survival curve comparison (RFS and OS). Dashed line highlights RFS or OS median for each subgroup of patients. Risk table was indicated for each Kaplan–Meier plot to show the number of patients at risk by time, for each subgroup. Hazard ratio (HR), confidence interval of HR (CI) and *p* value are evaluated with Univariate Cox regression model in RFS and OS. pTNM pathology tumor–node–metastasis.

Prognostic significance for OS of MIX scoring is weakly supported by survival curve (*p* = 0.13; Supplementary Fig. S4A and Fig. 6b) and univariate Cox model (HR: 1.3 with 95% CI 0.91–1.95, *p* = 0.136; Fig. 6a). Noted that sex and age have not a significant impact on survival rates (*p* = 0.24 and *p* = 0.457, respectively). However, OS analysis demonstrate that late stage (III/IV) is a poor prognosis factor (HR: 2.8 with 95% CI 1.9–4.05, *p* = 6.6 × 10^−8^; Supplementary Fig. S4A and Fig. 6b). Since multivariate analysis revealed that late stages and a high-MIX score were independent prognosis factors of patients’ outcome (Supplementary Fig. S4B), we chose to analyze prognostic value of MIX-staining on early stage and late stage, separately. Thus, survival curves highlight that high MIX staining is a bad prognosis factor on early stage of NSCLC (*p* = 0.016; Supplementary Fig. S5A, B) but is not informative to the prognosis of late stage (*p* = 0.91, *data not shown*). High MIX staining in early stage harbor a hazard ratio of 2.1 (95% CI: 1.13–3.83, *p* = 0.018, *data not shown*). To accurately estimate the prognosis value of MIX staining according to given stages, survival analysis (Kaplan–Meier and univariate Cox regression) according to Low or High MIX subpopulations at early (I/II) or late (III/IV) stages, were performed (Fig. 6b). MIX-High staining could be clearly considered as a poor prognosis marker only early stage. Since Kaplan Meier curves were still performed on two populations of patients combined (HCL and AMSBio), we evaluated independently each population to eliminate any bias due to the sample size. When the curves corresponding to each cohort are analyzed separately, we confirmed the MIX staining might be useful as a prognosis value regarding overall survival in early stages in comparison to all stages and late stages (Supplementary Fig. S5A–I).

As the same way, we have conducted RFS analysis. Only sex and stage have a significant prognostic value for recurrence in NSCLC and MIX staining appeared as no informative (data not shown). Nevertheless, we have pursued our RFS analysis, according to Low or High MIX subpopulations at early (I/II) or late (III/IV) stages. We show that late stage patients with High MIX staining seemed to have a worse overall survival (*p* = 0.13; Fig. 6a) compared to early stage patients with low staining. Univariate Cox model confirm that high MIX staining is a bad prognosis factor for recurrence (HR: 2.1 with 95% CI: 1.04–4.14, *p* = 0.038; Fig. 6a).

These results highlight the potential of MIX staining as a marker of tumor aggressiveness that could complement the existing TNM classification. Interestingly, it could reflect the CSCs Burden in tumor samples and thus could be clinically valuable for predicting patient outcomes in early-stage lung adenocarcinoma and might help to treatment decisions by providing insight into relapse risk.

Multiplex immunohistochemistry analysis showed that there were no B lymphocytes (LB, in red) infiltrating the lung tumor tissue at any stages. CD8+ T cells (CD8+ T cells, in green) are significantly increased in the area in which the MIX positive cells are localized at late stages. However, CD8+ T cells significantly reduced in non-tumoral lung tissue *(p* = 0.0001; Supplementary Fig. S6A, B). Likewise, in early stages, CD8+ T cell infiltration was significantly decreased, despite the presence of MIX-positive cells associated with CSCs in the tumor (*p* = 0.0001; Supplementary Fig. S6A, B).

These results were supported by previous findings demonstrated that CSCs create a tolerogenic immune microenvironment, promoting their survival and resistance to conventional treatments such as chemotherapy [16]. Thus, despite the infiltration of numerous immune cells surrounding CSC in lung adenocarcinoma, these immune cells do not necessarily exert anti-tumor activities [17].

## Discussion

The role of CSCs in lung cancer is of prime importance, as they have been implicated in the initiation, progression, and recurrence of the disease [6, 18]. These cells population contribute to the heterogeneity of lung tumors and play a role in tumor resistance to conventional treatments [19]. Thus, CSCs detection is essential to assess tumor aggressiveness, prevent relapse and therefore to increase long-term survival rates. As no specific treatment has yet been developed to directly target CSCs, their early detection could improve patient management and the implementation of clinical trials aimed at eliminating CSCs. Currently, the lack of specific CSC markers limits their routine clinical detection [1]. Since, various studies demonstrated that commonly recognized CSC markers, such as CD133, were not exclusively expressed in CSCs from tumors [12, 20, 21]. In surgically resectable lung cancer, the CSCs research may generate new approaches to improve early diagnosis, prevent recurrence, and for the long-term control of extensive disease. Thus, developing efficient methods to detect specifically CSCs is essential for clinical applications and translational research.

In this context, our aim was to compare CSCs characterization and sorting between MIX and CD133 and then to detect accurately NSCLC stem cells in patient solid biopsies. Our approach is based on specific recognition of glycan motifs expressed by lung CSCs. Normal cells or differentiated tumor cells are not detected by lectins mixture required to identify glycan patterns.

The effectiveness and reliability of lectins combination has been already demonstrated to detect stem-like cells from whole cancer cell population in CRC [13]. Since NSCLC might harbor CSCs with unique surface markers and molecular drivers due to the CSCs heterogeneity, glycan signature has been modified in the lung specific mix [1, 21]. First results demonstrated that MIX-positive cells represented 1% of tumor cell subpopulation which are closely similar to the CSCs percentage and overexpressed both common CSCs-related genes and protein markers [7, 18, 22]. Strikingly, the percentage of CD133-expressed MIX+ cells was reduced in the different adenocarcinoma cell lines suggesting that MIX should enable to detect CSCs not currently identified by CD133+. MIX positive cells which are negative for CD133+ could represent a new subpopulation of CSCs. Indeed, MIX-positive cells sorted by FACS exhibited significantly higher clonogenicity properties, supporting their self-renewal capacity similar to CSCs. This finding correlates with increased tumor recurrence and poor clinical outcome. Likewise, chemoresistance and tumorigenicity are one of the key characteristics of CSCs, their assessment was carried out using both in vitro and in vivo experiments in three distinct adenocarcinoma cell lines expressing or not EGFR mutations [23]. The chemosensitivity experiment confirmed that MIX-positive subpopulations are endowed with conventional therapy resistance compared to MIX− cells. Chemoresistance of MIX+ cells is even higher than those of CD133+ sorted from A549 and H1975 cells. Furthermore, the percentage of MIX+ cells rises in A549 cells following cisplatin treatment (data not shown) suggesting these cells are resistant or spared by cisplatin treatment and their subpopulation is markedly enriched similar to CD133+ cells [20, 24]. The functional properties analysis of MIX+ sorted cells confirms their aggressiveness and tumorigenicity in vivo, as tumor number and volume are significantly increased compared with negative cells. Furthermore, these changes of cell aggressiveness are accompanied by phenotypic modifications such as the expression of gene signature related to EMT which was observed in all lung cell lines. These changes are considered a CSCs silent characteristic, suggesting their ability to spread and migrate to distant sites [25]. Consequently, MIX+ cells sorted exhibit CSC-like properties independently of mutated EGFR expression in lung cell lines. Although in vitro and in vivo experiments confirmed an enrichment of “stemness” properties within MIX+ cell population, we still had to prove its efficiency in detecting ex vivo CSCs glycosylation from lung tumor patients. This step was necessary because previous studies, including one that examined 145 cases of stage I non-small cell lung cancer (NSCLC), revealed that CD133 overexpression alone was not a reliable predictor of recurrence or overall survival, in line with earlier study [26, 27]. The correlation between MIX staining and patient survival was to be verified. Indeed, the CSCs presence in tumor embedded section could be useful to discriminate tumor from healthy samples. Thus, the MIX sensitivity and specificity to detect NSCLC have been estimated by immunohistochemistry and showed a high specificity (100%) and a sensitivity around 72%. Contrary to the general association of poor prognosis factors with late-stage cancer, our results suggest that high scores are rather a poor prognostic factor in early-stage NSCLC. These data are of prime significance as even though NSCLC is diagnosed at early stages, almost a quarter of patients develop relapse and die from recurrent disease [28]. Indeed, it’s clearly recommended to emphasize on the characterization and to develop prognostic factors at early stage [29]. Furthermore, we showed that T cytotoxic Lymphocytes infiltrated the tumor since early stages. However, their anti-tumor immune response should be inhibited by the presence of CSCs which induces a tolerogenic immune microenvironment and promotes the chemotherapy resistance [16]. This association between CD44, a CSC marker and immune cells was already considered as a negative prognostic factor [17]. In this context, the prognostic value of MIX score in early-stage NSCLC could enhance patient monitoring and reduce relapse risk.

Altered glycosylation is a hallmark of various cancers regardless of the origin and stages and aberrant glycosylation patterns are also a common features of CSCs population markers and signaling pathways [30, 31]. The comparison of MIX positive cells with CD133-positive ones allow to prove once upon a time that glycosylated patterns are determinant for cancer stem cells properties and detection. Nevertheless, the lectins combination appears to be more relevant than CD133 to detect and sort CSCs. Thus, LungSTEM MIX could enable personalized therapeutic strategies in the future. By adapting therapies based on the unique characteristics of CSC present in each patient, treatment outcomes can be optimized, potentially resulting in higher response rates, reduced relapse rates, and improved overall clinical outcomes [32].

## Conclusion

The identification of CSCs burden with the Lectins mix might afford a valuable companion prognostic tool in early stages NSCLC patients which might lead to the emergence of personalized therapeutic strategies targeting CSCs in the future. This study enables a deeper understanding of the lung cancer and might help to evaluate the risk of relapse. This new advancement should allow clinicians to better stratify patients and optimize treatment selection based on the presence of NSCLC stem cells, leading to better outcomes.

## Supplementary information

Supplementary Materials and Figures

## Data Availability

Data generated or analyzed during this study are included in this published article and its supplementary files. The data that support the findings of this study are also available upon request from corresponding authors.
