# Supplementary material for: Comparison of methods for cancer stem cell detection in prognosis of early stages NSCLC
Source: Br J Cancer. 2024 Sep 20;131(9):1425–36. doi: 10.1038/s41416-024-02839-9 (PMC11519646; doi:10.1038/s41416-024-02839-9)
Supplement: Supplementary file 1 — Supplementary Materials and Figures [file 41416_2024_2839_MOESM1_ESM.pdf]

Supplementary Materials

Indirect Magnetic Cell Sorting

MACS sorting was performed from 2.10^7 cells/mL using the CELLection™ Biotin Binder kit (Invitrogen-ThermoFisher Scientific, France) according to the manufacturer's instructions, by using 10 µg of either the MIX (LungSTEM) or the AC133 biotinylated antibody (Miltenyi Biotech, France). For the clonogenic assay, sorted cells were seeded in ultra-low attachment 96-well plates (Falcon Corning brand, France) at decreasing cell densities (1000, 100, 10, and 1 cell) to evaluate their clonogenic potential.

Clonogenicity Assay

In each experimental condition sorted cells were seeded in triplicate in a defined medium. Weekly, 50 microliters of the medium were added to each well, and this process continued for a period of 4 to 8 weeks. The quantification of sphere formation was performed per well and per condition, employing an optical microscope (Olympus CKX53, Life science, Waltham, Massachusetts) at a magnification of x100. The spheroids were consistently imaged at regular intervals, precisely every 7th day (Day+7). The measurement of spheroid size was accomplished using ImageJ software.

Cell Viability Assay

For each condition, 1500 cells were sorted and seeded in a 96-well plate. Cells were treated, 24h later, with increasing doses of Cisplatin (0 to 200 µM). Following 72h of incubation, the Cell Viability Kit reagent (Promega, USA) was added and luminescence measured with EnSpire® Multimode Plate Reader (PerkinElmer, USA).

Multiplex immunohistochemistry for immune microenvironnement analysis

The multiplex immunohistochemistry was done on 4 µm paraffin-embedded histological sections using the SignalStar™ Spatial Profiling kit (cell signaling, US) according to manufacturer's instructions. The sections were stained with CD8α (D8A8Y) & CO-0004-488 SignalStar™ Oligo-Antibody Pair (85336, Cell Signaling), CD3ε (D7A6E™) & CO-0001-647 SignalStar™ Oligo-Antibody Pair (33888, Cell Signaling) CD20 (E7B7T) & CO-0011-594 SignalStar™ Oligo-Antibody Pair (54189, Cell Signaling) and nuclei were counter-stained with Dapi (Sigma) and the slides were scanned with the NanoZoomer RS 2.0 Hamamatsu (Hamamatsu Photonics, Massy, France).

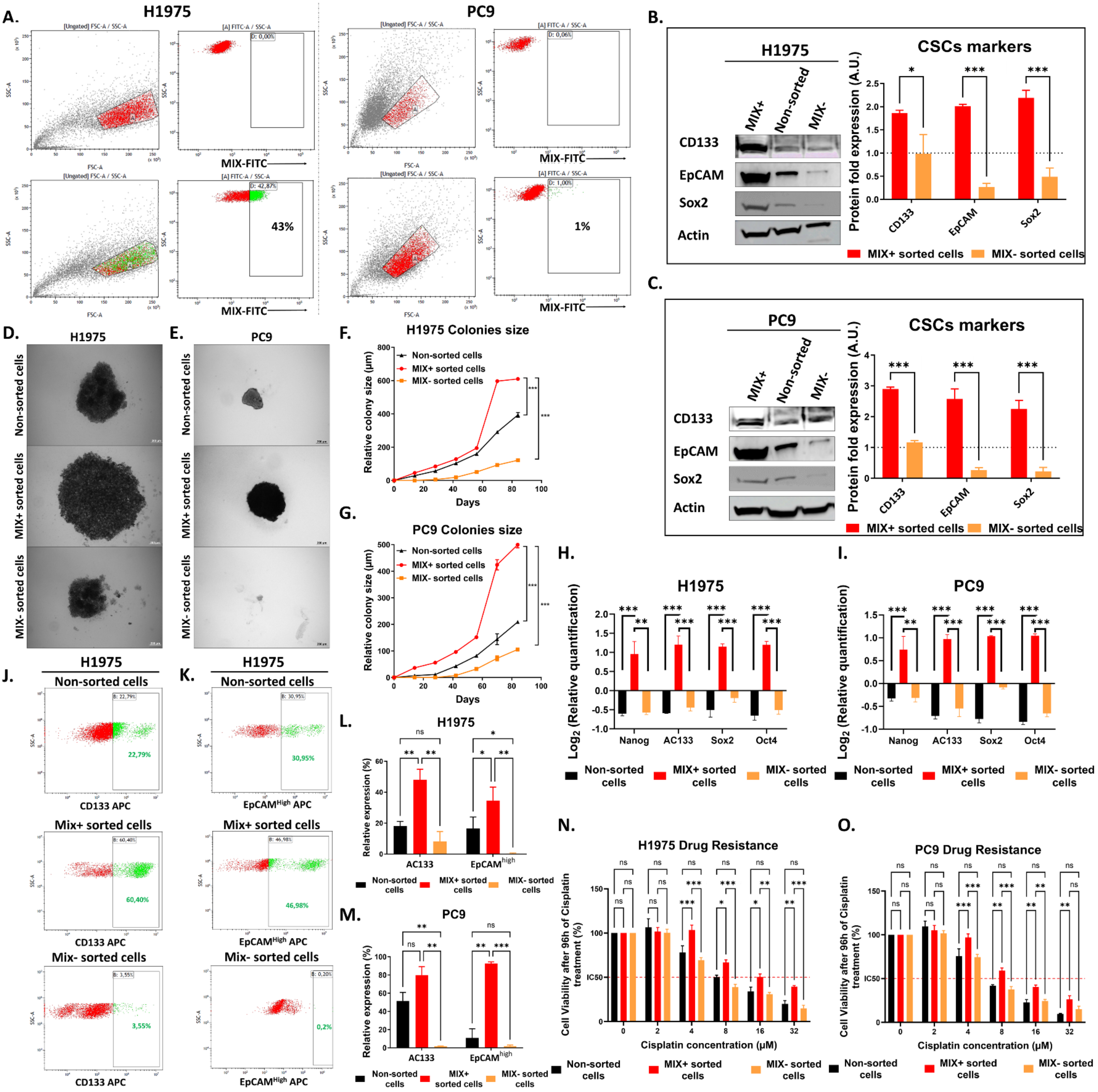

Supplementary Figure S1. Analysis of stemness characteristics and properties in MIX-sorted cells from H1975 and PC9 lung cancer cell lines

(A) Representative FACS dot plots (top) showing the recognition of glycosylated patterns by the isotypic (diluent) control condition. Bottom, expression of glycosylated patterns detected by the LungSTEM MIX in both H1975 (at left) and PC9 (at right). (B) Western Blot shows the cancer stem cell related proteins expression (CD133, EpCAM, Sox2, Actin) in each sorted or non-sorted sub-populations (MIX+, non-sorted cells and MIX-), at left, and its corresponding quantification normalized to non-sorted cells out of three replicates (at right) in H1975 cell line.

(C) Similar results are shown in PC9 cell line. (D, E) Clonogenic capacity after FACS single cell sorting. Representative self-renewal ability in different sorted sub-population are depicted, from MIX+ or MIX- cells or Non-sorted cells in both H1975 (D) and PC9 (E) cell lines (magnification x100). (F, G) Relative colonies sizes of MIX+ and MIX- sorted cells compared with control non-sorted cells after FACS single cell sorting in H1975 (F) and PC9 (G) cell lines. The spheres' size was monitored by recording pictures every D+7 for 80 days. (H, I) Representing mRNA expression levels of cancer stem cell genes (Nanog, AC133, Sox2 and Oct4) in different sorted sub-population (MIX+ or MIX-) or non-sorted one (Non-sorted cells) both in H1975(H) and and PC9 (I) cell lines. (J, K) Representative flow cytometry dot plots of relative expression of AC133 (J) and EpCAMhigh (K) after FACS single cell sorting with LungSTEM MIX compared with non-sorted cells in H1975 cell line. Similar experiments were done for PC9 (Data not shown). (L, M) Histogram representing EpCAMhigh and AC133+ percentages analyzed by Flow cytometry within MIX+, MIX- and Non-sorted cells in both H1975 (L) and PC9 (M) cell lines. (N, O) Assessment of drug resistance to cisplatin. Histogram representing cell viability upon 96h Cisplatin treatment at different concentrations on MIX+, MIX- and non-sorted cells after FACS on 1500 cells seeded per well in both H1975 (N) and PC9 (O) cell lines. Results are represented as mean ± SEM, **ns** not significant result, * p-value < 0.05; ** p-value < 0.01; *** p-value < 0.001 using one-way ANOVA test (n=3 experiments) or student test (n=3 experiments).

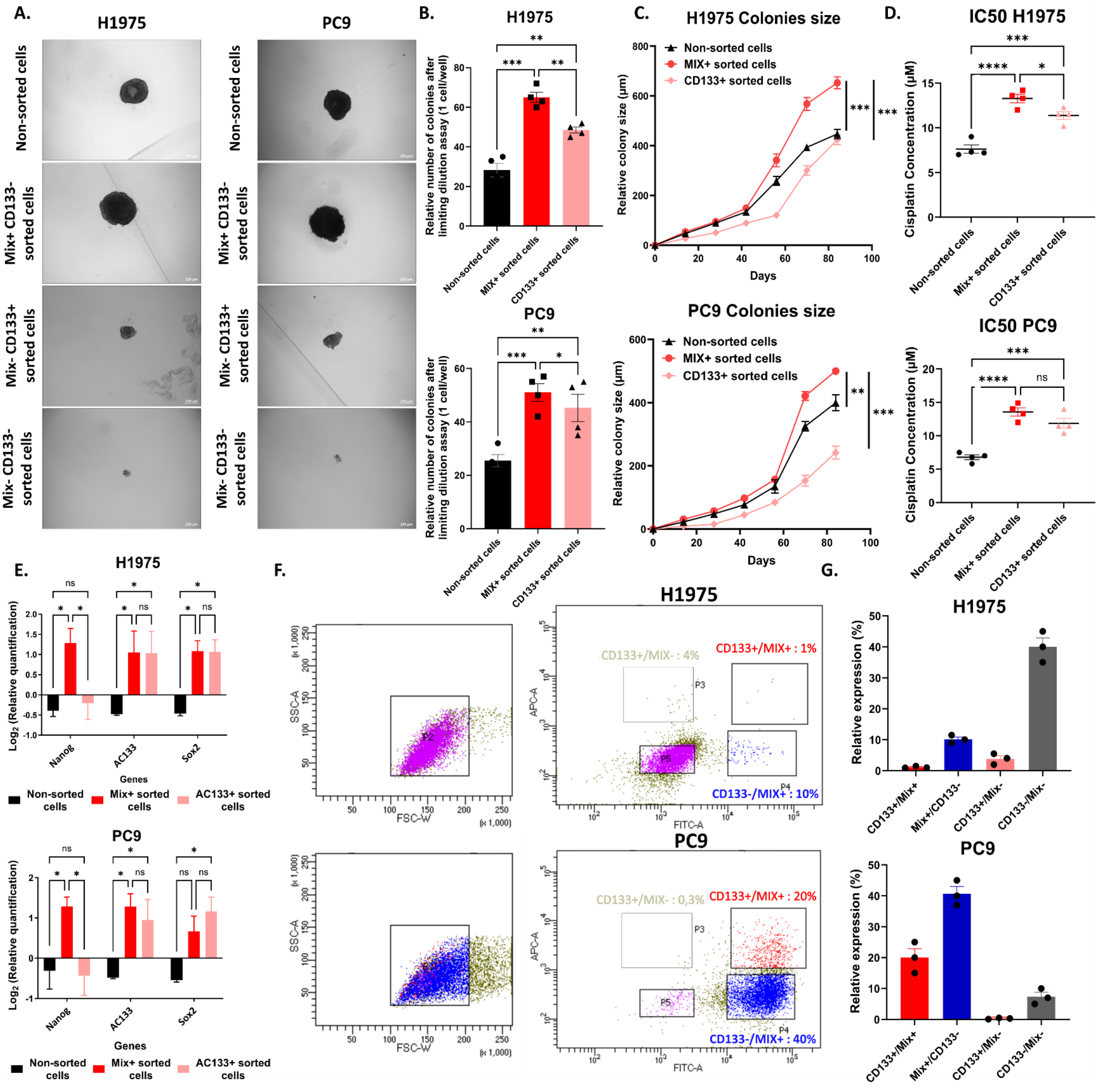

Supplementary Figure S2. In vitro comparison between LungSTEM MIX and CD133 efficiency in lung CSCs detection and characterization.

A. Clonogenic capacity after Limiting Dilution Assay. Representative sphere forming ability in different sorted sub-population are depicted, from AC133 (AC133+ and AC133- sorted-cells compared with Non-sorted cell, at left) and lungSTEM MIX (MIX+ and MIX- sorted cells

compared with Non-sorted cell, at right), according to seeded cell densities (1, 10, 100, 1000 cells/well) (magnification, x100). B. Histograms represent the mean of sphere number formed after Limiting Dilution Assay in the condition with 1 cell/well. C. Relative colony size in condition of 1 cell/well following Limiting Dilution Assay in different sorted sub-population (AC133+, MIX+) compared to non-sorted cells. D. Cell viability upon increasing concentrations of cisplatin treatment after MACS cell sorting on AC133 and LungSTEM MIX to assess drug resistance in MIX+, AC133+ and Non-sorted cells. E. Representation of the average IC50 of Cisplatin (µM) in the different sorted (MIX+, AC133+) and Non-sorted cells. F. Analysis of CSCs genes levels (Nanog, AC133, Oct4, Sox2) in different sorted sub-population (MIX+, AC133+) and Non-sorted cells. G. Representative FACS dot plots showing the basal expression of AC133 (at left), LungSTEM MIX (at middle) and coexpression (at right) in Non-sorted cells H. Representative FACS dot plots showing AC133 expression (at left), LungSTEM MIX expression (at middle) and coexpression of AC133 and MIX (at right) in Mix+ sorted cells. I. Representative FACS dot plots showing AC133 expression (at left), LungSTEM MIX expression (at middle) and coexpression of AC133 and MIX (at right) in AC133+ sorted cells. All results are represented as mean ± SEM, **ns** indicate not significant result, * p-value < 0.05; ** p-value < 0.01; *** p-value < 0.001 using one-way ANOVA test (n=3 to n=4 experiments).

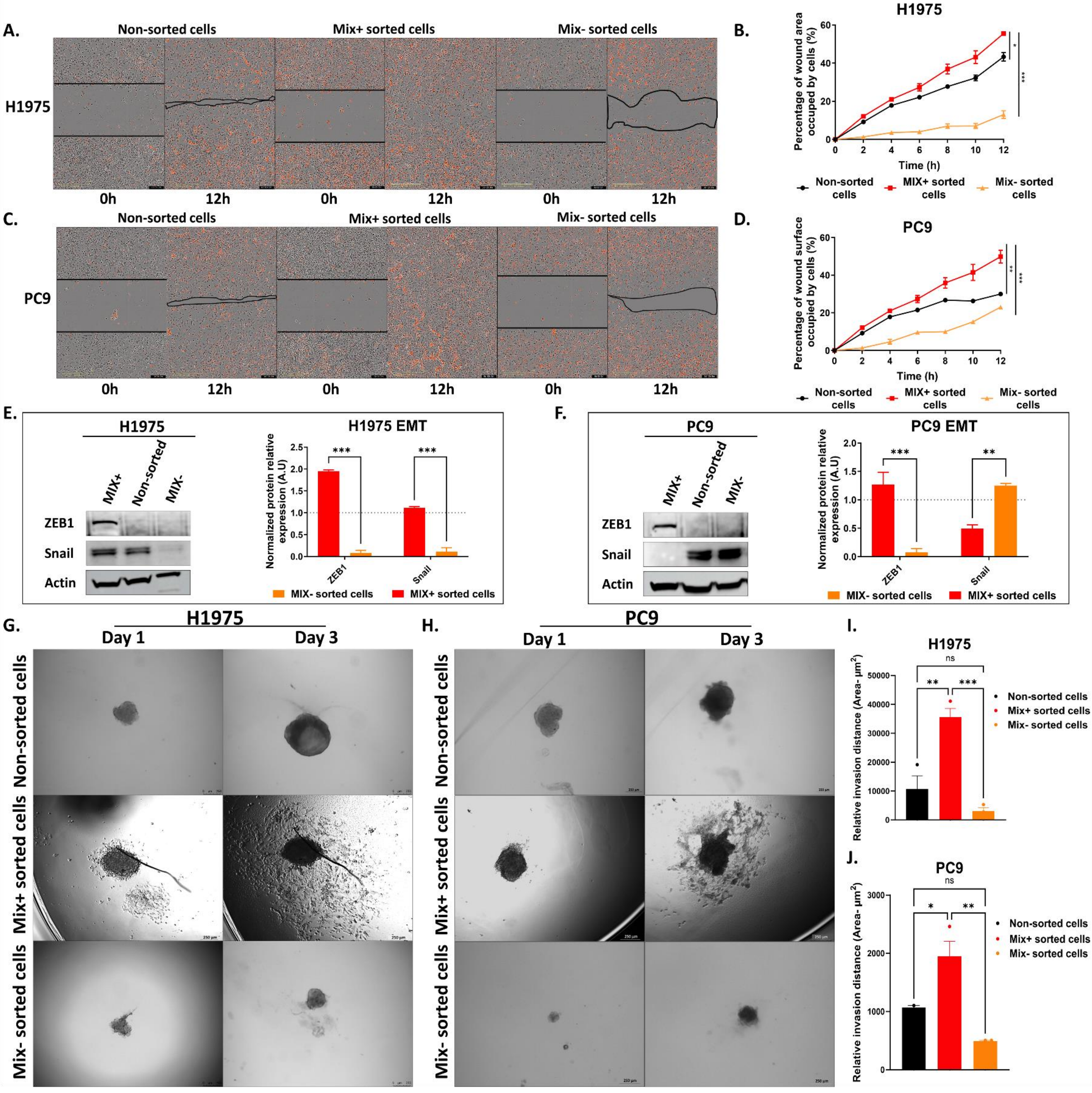

Supplementary Figure S3. Involvement of MIX+ stem cells like in the migration and invasion of H1975 and PC9 cell lines

Wound healing scratch assay is performed using Incucyte (magnification x10) with mutated EGFR cell lines, H1975 (A) and PC9 (C) following MIX cell sorting (MIX+ and MIX-) or not (non-sorted cells). Curves showing the percentage of wound area occupied by cells were measured using Incucyte 2022 Rev1 Software in H1975 cell line (B) and PC9 cell line (D). Western Blot shows Epithelial-mesenchymal transition (EMT) related proteins levels in each sorted subpopulation (MIX+ and MIX-) or non-sorted cells in H1975 (E) and PC9 (F) cell lines (left panel) and quantifications normalized to non-sorted cells out of three replicates (right

panel) in both cell lines. (G, H) Representative images of spheroids' invasion capacities after 3 days of incubation in Matrigel coated inserts, in each sorted sub-population (MIX+, MIX-) and non-sorted cells from H1975 and PC9 cell lines. (I, J). Histogram representing the relative invasion capacity of spheroids from each sorted subpopulation (MIX+ and MIX-) and in unsorted cells. Spheroids from each subpopulation were embedded in Matrigel. Matrigel invasion was measured by deducting the total area from the central area, using the Fiji Macro analysis program. Results are represented as mean ± SEM, **ns** for not significant result, * p-value < 0.05; ** p-value <0.01; *** p-value < 0.001 using one-way ANOVA test (n=3 experiments) or t-test (n=3 experiments).

A.

		Univariate Cox analysis			Multivariate Cox analysis		
		HR	95% CI of HR	P Value	HR	95% CI of HR	P Value
Sex	Male (ref.)						
	Female	0.8012	0.5534 - 1.16	0.24	/	/	/
Age	≤ 60 yrs (ref.)						
	> 60 yrs	1.148	0.7976 - 1.654	0.457	/	/	/
Stage	Early (ref.)						
	Late	2.791	1.923 - 4.051	**6.57e-08**	2.838	1.942 - 4.146	**7.01e-08**
MIX staining	Low (ref.)						
	High	1.334	0.9133 - 1.948	**0.136**	1.382	0.933 - 2.047	**0.107**

B.

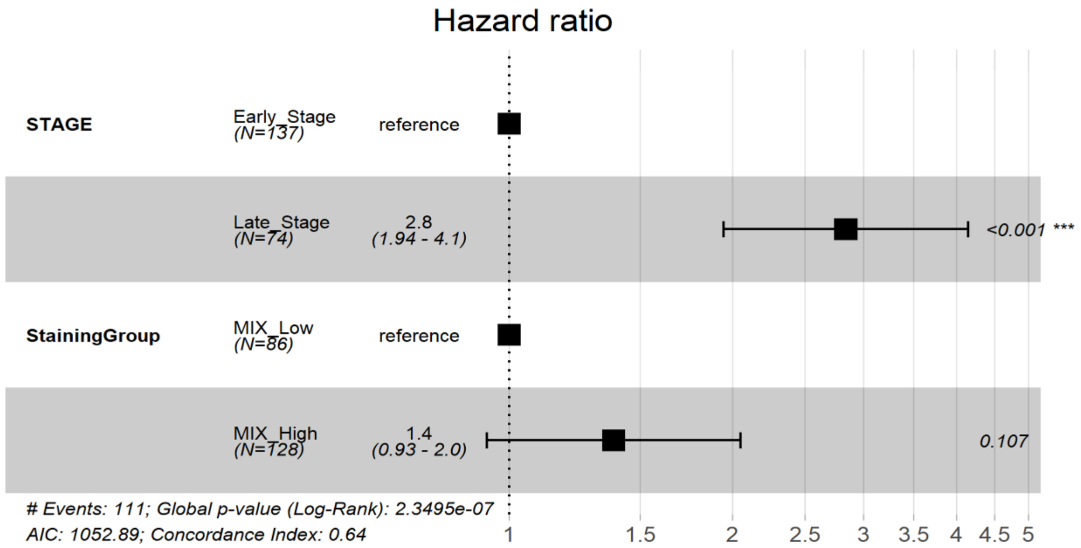

Supplementary Figure S4. Prognostic values of clinicopathological features (gender, age, stage and LungSTEM (MIX) staining) at the lung adenocarcinoma patient's follow-up

A. Results of the univariate and multivariate Cox regression analyses regarding overall survival (OS) in the cohort. Multivariate analysis was performed with features harboring a p value inferior to 0.2 in univariate analysis. **B.** Forest plot of Hazard Ratio (HR) for OS by Cox multivariate analysis. *HR, Hazard ratio; C.I., Confidence Interval*.

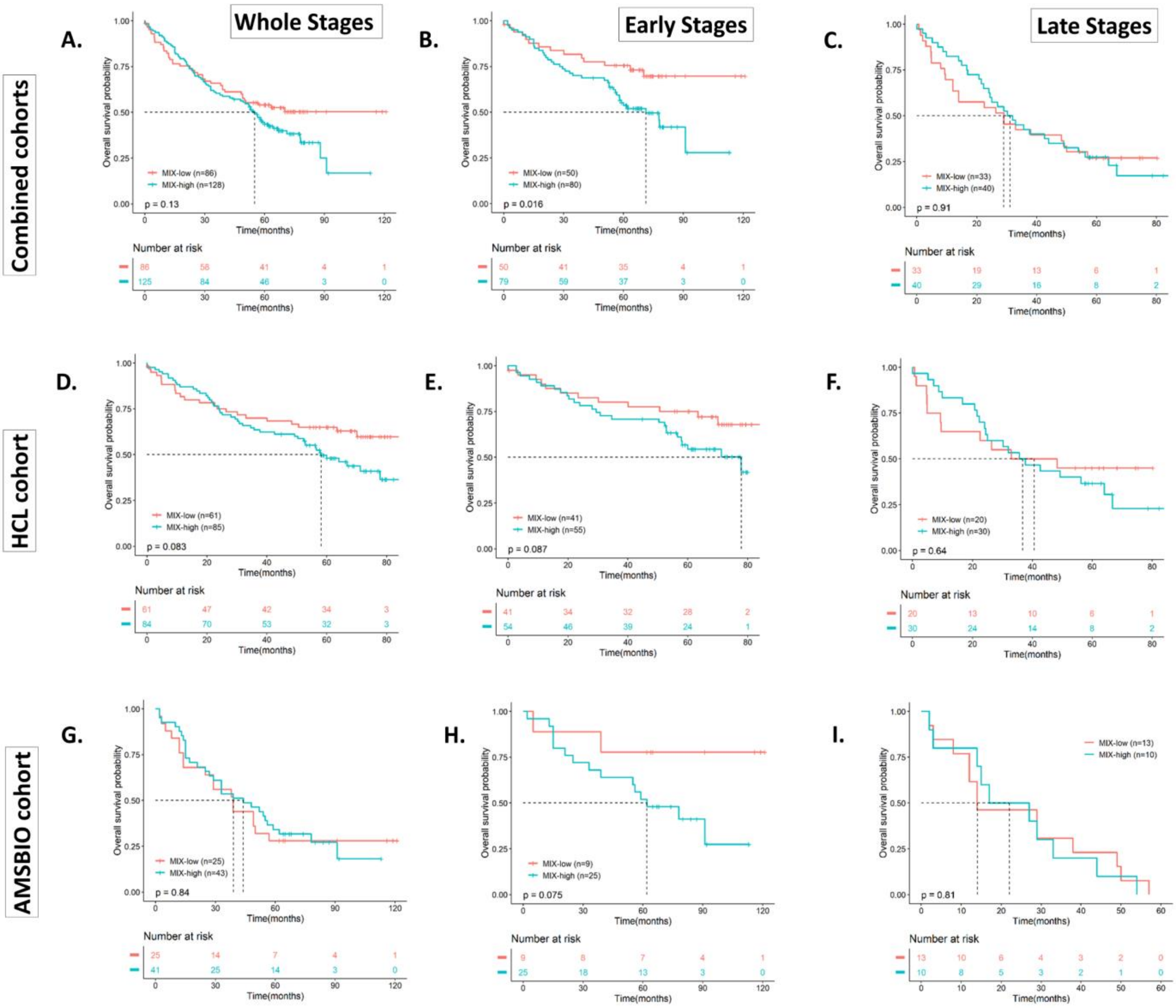

Supplementary Figure S5: MIX staining contribution in the prognosis evaluation (overall survival) at any stages, early stages (I and II) and late stages (III and IV)

Kaplan-Meier curves are depicted according to MIX-Low versus -High staining, regardless of the stage of lung adenocarcinoma (A, D and G) or only on the early stages (B, E and H) or late stages (C, F and I). Each analysis was performed three times according patient cohorts, i.e combined cohort or two independently cohorts (HCL and AMSBIO). Top panels show survival curves established from combined cohort (A, B and C) and below panels shows survival curves established from two independantly cohorts, HCL cohort (D, E and F) and AMSBIO cohort (G, H and I). P-value indicated in each panel correspond to log-rank test (Mantel-Cox) performed to survival curves comparison (overall survival). Dashed line highlights median OS for each subgroup of patients, according to MIX staining. Risk table was indicated for each Kaplan-Meier plot, to show the number of patients at risk by time, for each group according MIX staining.

A.

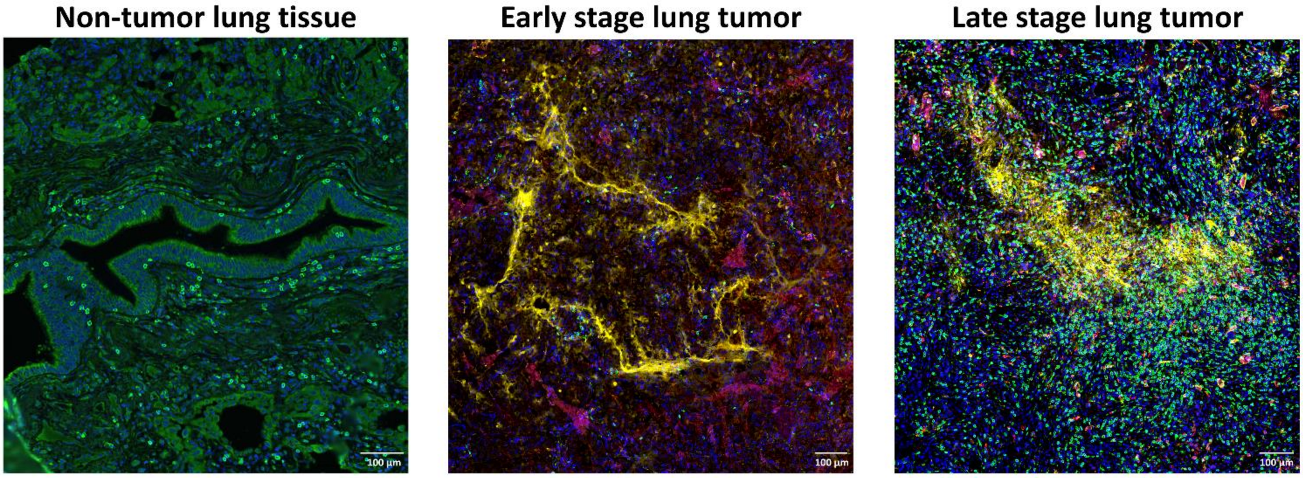

B.

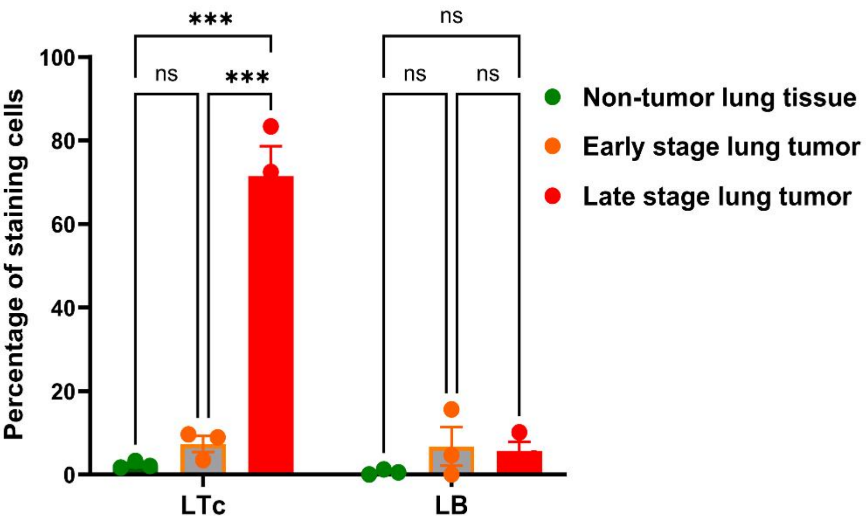

Supplementary Figure S6: LungSTEM MIX and immune microenvironment expression in non-tumor and tumor lung tissues from early and late stage patients

A. mIHC of NSCLC FFPE sections of non-tumor lung tissue (at left), early stage lung tumor (at middle) and late stage lung tumor (at right) labelled with DAPI (blue), MIX positive cells (yellow), CD8α (green) marker for cytotoxic T Lymphocytes, CD20 (red) for B lymphocytes, FoxP3 (cyan) for CD4+ regulatory T cells, CD3ε (magenta) for T lymphocyte were scanned using the NanoZoomer RS2 Hamamatsu imaging system (magnification, 200x). **B.** Histogram representing percentage of T (left) and B (right) lymphocytes counted in each FFPE sections. Experiments were done on 10 sections and results are represented as mean ± SEM, **ns** for not significant result, *** p-value < 0.001 using one-way ANOVA test. *mIHC/IF*: multiplex immunohistochemistry/immunofluorescence; *FFPE*: Formalin-Fixed Paraffin-Embedded; DAPI: 4′,6-diamidino-2-phenylindole.
